# Causal correlation paths across cortical areas in decision making

**DOI:** 10.1186/1471-2202-15-S1-O7

**Published:** 2014-07-21

**Authors:** Adrià Tauste Campo, Marina Martinez-Garcia, Verónica Nácher, Gustavo Deco, Ranulfo Romo

**Affiliations:** 1 Department of Information and Communication Technologies, Universitat Pompeu Fabra, Barcelona, 08018 Spain; 2 Instituto de Fisiología Celular-Neurociencias, Universidad Nacional Autónoma de México, 04510 México D.F., México

## 

We study how neural spike activity encode, integrate and communicate information across different brain areas. An ideal paradigm to study this problem is the vibrotactile discrimination task designed by Romo *et al. *[1]. This is a complex process, which requires communicating information from the sensory areas that perceive the tactile stimuli to superior areas that integrate this sensory information and report the decision. Previous works on this task have characterized the role played by sensory and motor areas using the correlation between single-neuron rate responses and the task variables, namely the two stimulation frequencies and the decision [2]. In the present work, we investigate the causal correlations that arise between nearby and distant cells while the monkey is performing the task under fixed stimulation frequencies.

To this end, we use simultaneous multiple-cell recordings to estimate causal across five cortical areas (S1, S2, SMA, DPC and M1) over the time course of the discrimination task. Causal correlations are estimated with a sequential universal estimator of the directed information based on the context-tree weighting algorithm [3,4]. Statistical tests on the estimates for four stimulation frequency pairs suggest that significant causal correlations (‘causal paths’) are highly distributed across the studied cortical areas and are equally present in feedforward and feedback interactions between sensory and motor areas. Furthermore, the percentage of incoming causal paths is steady during the time course of the task for destination areas S2, SMA, DPC and M1 while it decays during the stimulation periods for S1. The task-specificity of these results is assessed by a control task, where the monkey receives both stimuli but it is requested not to perform the task. Specifically, during the passive stimulation task there is an abrupt decrease in the number of causal correlations after the first stimulation, which is shown to be independent of the spike-train variability of each area.

## Conclusions

Neuronal causal correlation paths that are specific to the discriminations task are ubiquitous, bidirectional and remain approximately constant along the task in both sensory and motor areas. These findings are robust to the stimulation pair under study and the spike-train variability of each area.
